# Herbivory by Leaf-Cutting Ants: Exploring the Jasmonate Response in Host and Non-Host Plants

**DOI:** 10.1007/s10886-024-01519-5

**Published:** 2024-06-20

**Authors:** Andrea Teresa Müller, Kilian Lucas Ossetek, Axel Mithöfer

**Affiliations:** Research Group Plant Defense Physiology, Max Planck Institute for Chemical Ecology, Jena, Germany

**Keywords:** *Atta*, Herbivory, Jasmonates, Plant Volatiles, *Spodoptera*, Wounding

## Abstract

Leaf-cutting ants (Formicidae; *Atta* spp., *Acromyrmex* spp.) cut off pieces of leaves and other plant tissue and feed it to their symbiotic fungi. As this foraging behavior poses an imminent threat to agriculture, leaf-cutting ants are considered as pests of huge ecologically and economically importance. Consequently, research on leaf-cutting ants focused on their foraging decisions and interactions with their cultivated symbiotic fungi, whereas their effect on the attacked plants, apart from the loss of plant tissue, remains largely unknown. In this study, we investigated the consequences of an attack by leaf-cutting ants and analyzed the plants’ defense responses in comparison to chewing caterpillars and mechanical damage. We found that an attack by leaf-cutting ants induces the production of jasmonates in several host and non-host plant species (*Arabidopsis thaliana*, *Vicia faba*, *Phaseolus lunatus*, *Tococa quadrialata*). Additionally, we showed in the natural host plant lima bean (*P. lunatus*) that leaf-cutting ant damage immediately leads to the emission of typical herbivory-induced plant volatiles, including green leaf volatiles and terpenoids. Further data exploration revealed clear differences in the defense-related phytohormone profile in plant species of Neotropical and Eurasian origin. Taken together, we show that leaf-cutting ant infestation and their way of clipping the plants’ tissues induce jasmonate and jasmonates-mediated responses and do not differ from those to mechanical injury or larval feeding.

**Supplementary Information:**

The online version contains supplementary material available at 10.1007/s10886-024-01519-5.

## Introduction

Leaf-cutting ants (Formicidae; Myrmicinae; tribe Attini; genera *Atta*, *Acromyrmex*) are widely distributed in arid, semi-tropical and tropical regions of the Americas, including native and anthropic ecosystems (Wirth et al. 2003). In contrast to the dominating omnivorous *Camponotus* spp., leaf-cutting ants are polyphagous herbivorous insects and belong to the ecologically most dominant ant species everywhere they are found (Wirth et al. 2003). Foraging leaf-cutting ants cut off pieces of leaves and other plant tissues, thereby removing up to 20% of the leaf material of individual plants from up to 50% of the plants in the neighborhood of the colonies (Cherrett 1968; Wirth et al. 2003). Strikingly, these ants do not feed directly on the harvested plant material but carry it to their nests to cultivate symbiotic fungi, which in turn provide protein-rich nutrition for the ants. While the ant larvae completely rely on fungal food, the adult ants also feed on plant sap (Littledyke and Cherrett 1976). Due to their lifestyle, in agricultural and silvicultural areas, they are important pests that cause immense economic damage (Montoya-Lerma et al. 2012). Therefore, leaf-cutting ants are probably among the most studied tropical insects. As indicated by Wirth et al. (2003), the focus of those studies was either on pest control (Vander Meer et al. 1990; Della Lucia et al. 2014), ant-fungus interactions (Weber 1966; Caldera et al. 2009), foraging strategies (Cherrett 1968; Roces 2002), or chemical and physical aspects for host plant selection (Vasconcelos and Fowler 1990).

Although leaf-cutting ants attack many different plants, at any given time most of the harvested material comes from only a few species (Cherrett 1968; Wirth et al. 2003). For example, studies of *Atta cephalotes* L. have shown that host plant selection is closely related to plant secondary chemistry. In particular, terpenoids or non-identified lipids have been described to repel these ants or to deter the harvest of otherwise acceptable substrates (Littledyke and Cherrett 1978; Chen et al. 1984; Hubbell et al. 1983; Howard 1988; Howard et al. 1988). All these studies were conducted with compounds that are constitutively present. What has been mainly neglected up to now is the question of how the host plants react to the leaf-cutting ants’ attack on demand. Leaf-cutting ants stop exploiting a host plant long before it has been defoliated completely (Cherrett 1968; Wirth et al. 2003). The reason behind this is unknown, but could be explained by the fact that continuous removal of leaf material from a plant, in other words herbivory, may lead to changes in the chemistry of the host due to the induction of anti-herbivore defenses. Such herbivory-induced plant defenses are very well known (Mithöfer and Boland 2012).

Strikingly, there are only few studies in the literature dealing with the analysis of leaf-cutting ant-induced defenses. *Acromyrmex rugosus* F. Smith herbivory induced changes in leaf trichome density and changed the emission of volatile organic compounds (VOC), mainly terpenoids, in the aromatic plant *Ocimum gratissimum* L. (Tozin et al. 2017). Kost and coworkers (2011) showed that *Phaseolus lunatus* L. leaves exposed to *Atta colombica* Guérin-Méneville also emitted more VOC than control plants when the leaf damage was stepwise increased over a longer period. Induced VOC release as well as the accumulation of defensive compounds and defense-related genes typically depends on jasmonate phytohormones. Jasmonates are primarily responsible for the induction of plant defenses upon insect herbivory (Gatehouse 2002; Mithöfer and Boland 2012).

Although in the mentioned study (Kost et al. 2011) a role for the wounding- and herbivory-induced jasmonates upon *A. colombica* herbivory was suggested, these endogenous signaling compounds or any other defense-related responses have not been analyzed, neither in this nor in other studies. The recognition of herbivory and the downstream induction of jasmonates is necessary for the initiation of defense responses. A herbivore that can feed largely or completely undetected could be extremely successful and may have a drastic impact, at least on the vegetation in the vicinity – as in the case of leaf-cutting ants.

In this study, we hypothesized that plants under leaf-cutting ants attack can respond accordingly by jasmonate mediated defenses. Thus, we analyzed jasmonates in plant leaves of four different species upon leaf-cutting ant attack in comparison with mechanical wounding and feeding of an herbivorous chewing insect larva. To further investigate whether observed responses to Neotropical leaf-cutting ants may depend on a common regional origin, we included two Eurasian non-host plants and two Neotropical host plants in the study.

## Methods and Materials

### Plants and Growth Conditions

All plants were raised from seeds. Lima bean (*P. lunatus;* Fabaceae) and faba bean (*Vicia faba* L.; Fabaceae) plants were cultivated in a growth chamber under long-day conditions (16 h/8 h light/dark cycle), Arabidopsis (*Arabidopsis thaliana* (L.) Heynh.; Brassicaceae) Col-0 plants under short-day conditions (10 h/14 h, light/dark cycle). Relative humidity was 50–60% at 21 °C and light intensity was 100 µmol m^− 2^s^− 1^. Experiments were performed on 10-d old *P. lunatus*, 3-week-old *V. faba*, and 5-6-week-old *A. thaliana* plants, respectively. Tococa (*Tococa quadrialata* (Naudin) J. F. Macbr., recently renamed *Miconia microphysca* Michelang.; Melastomataceae) plants were grown in a glasshouse under long-day conditions (16 h/8 h light/dark cycle) at 23–25 °C/16–18 °C (day/night) and a relative humidity of approx. 70%. Experiments were performed with mature plants (ca. 30 cm tall) according to Müller et al. (2024). The origin of tococa and lima bean are the Neotropics. It is well known that in the field both Neotropical plant genera are natural hosts of *Atta* species (Vieira et al. 1992; Michelangeli 2003; PlantwisePlus Knowledge Bank 2022).

### Herbivory Experiments

All experiments took place under controlled conditions. For experimental details such as sample size, time periods or the particular statistical method used, see the respective legends. Arabidopsis, lima bean, faba bean and tococa plants were randomly assigned to four groups. Plants of the first group were exposed to an *A. cephalotes* ant colony for 1 h (Arabidopsis, lima bean, faba bean) or 3 h (tococa; due to leaf thickness it took longer to get the same level of wounding), where the ants were allowed to move around freely and cut off leaf snippets that accounted for 20–35% of the total leaf area. These ants were raised in an artificial environment and were exposed to *Rubus* sect. *Rubus* (Rosaceae) leaves before the start of the experiment, as pre-exposure to potential harvesting material enhances their leaf cutting activity (pers. observation). The *A. cephalotus* colony was 11 years old with about 100,000 individuals. The second group of plants was exposed to mechanical damage mimicking the wounding caused by the ants. For 1 h (Arabidopsis, lima bean, faba bean) or 3 h (tococa), pieces were excised from the leaves of the plants in a manner and frequency similar to the ants. More precisely, we studied the ants’ excision strategy (which parts of the leaf were cut out, the sizes of the leaf pieces, time to cut out a piece, time between cuts) and mimicked it manually with scissors. Herbivore damage by a generalist caterpillar served as a positive control. Therefore, *Spodoptera littoralis* Boisduval (Lepidoptera, Noctuidae) larvae were hatched from eggs and reared as described in Müller et al. (2024). Second and third instar larvae were chosen for the herbivory experiments and starved 24 h prior to plant feeding. Insects were placed on each plant of the group and allowed to feed on the leaves for 1 h (Arabidopsis, lima bean, faba bean) or 3 h (tococa). One group served as negative control and did not receive any treatment. In addition, the experiment with Arabidopsis and leaf-cutting ants was repeated with a new batch of Arabidopsis plants and a colony of *A. sexdens* ants instead of *A. cephalotes* (for the experimental approach and the resulting leaves after the experiment see Fig. S1). *A. sexdens* ants were also raised in an artificial environment and exposed to dried oat flakes (*Avena* spp.) prior to the start of the experiment. The colony was 10 years old with about 25,000 to 35,000 individuals. All treated leaves were excised immediately or 3 h (tococa) after the respective treatments, photographed and flash-frozen in liquid nitrogen. The samples were stored at -80 °C until further processing.

### Analysis of Plant Defensive Hormones

The frozen leaves of each plant were pooled and ground in liquid nitrogen using mortar and pestle. 100 mg of the resulting leaf powder were extracted with 1 mL of methanol containing 40 ng mL^− 1^ of D_6_-jasmonic acid (JA), D_6_-abscisic acid (ABA) and 8 ng mL^− 1^ D_6_-JA-Isoleucine conjugate (JA-Ile) (HPC Standards GmbH, Borsdorf, Germany). The homogenate was mixed for 30 min and the debris removed by centrifugation (10 min; 16,000 g). The defensive hormones JA, JA-Ile, ABA, OH-JA, OH-JA-Ile, and COOH-JA-Ile were analyzed by LC-MS/MS using an LC-TripleQuad-MS system as described in Davila-Lara et al. (2021) (Arabidopsis, lima bean, faba bean) and Müller et al. (2022) (tococa). Compounds were quantified by comparison of the sample peak areas to the peak area of the corresponding internal standards. The D_6_-JA-Ile standard was used for JA-Ile, COOH-JA-Ile, and OH-JA-Ile.

### RNA Extraction and qPCR

The frozen leaves were ground in liquid nitrogen using mortar and pestle. Sample lysis was achieved by adding 1 mL of TRIzol (Thermo Fisher Scientific Inc., Darmstadt, Germany) to 100 mg of leaf powder, homogenization and further incubation for 20 min at room temperature. For the removal of DNA and proteins, 300 µL of CHCl_3_ (Carl Roth GmbH, Karlsruhe, Germany) were added to each sample. All samples were mixed and incubated on ice for 20 min before the phenol-chloroform phase and the aqueous phase were separated by centrifugation (4 °C, 30 min; 16,000 g). The upper aqueous phase containing RNA was transferred to a new tube. To precipitate the RNA, 600 µL of isopropanol (Carl Roth GmbH) were added to the solution and the mixture was incubated overnight at -20 °C before centrifugation (4 °C, 30 min; 16,000 g) separated the precipitated RNA. The pellet was washed twice with 80% ethanol. Any residual solvent was removed using a concentrator plus (Eppendorf AG, Hamburg, Germany), before the RNA was re-dissolved in 80 µL RNAse-free water (preheated 60 °C) and stored at -80 °C until further usage.

RNA samples were subjected to DNA digestion using the TURBO DNA-free kit (Life Technologies, Carlsbad, CA, USA). From purified RNA samples cDNA was synthesized using the RevertAid First Strand cDNA Synthesis kit (Thermo Fisher Scientific Inc.). Quantitative real time PCR (qRT-PCR) was performed on the CFX96 Touch™ Real-Time PCR System (Bio-Rad Laboratories GmbH, Feldkirchen, Germany). Reactions were carried out in optical 96-well plates using the qRT-PCR Brilliant II SYBR Master Mix (Thermo Fisher Scientific Inc.) with 50 ng cDNA as template. PCR conditions were: initial denaturation: 3 min, 95 °C; 45 x (denaturation: 30 s, 95 °C; annealing: 30 s, 60 °C, extension: 30 s, 72 °C); final denaturation: 10 s, 95 °C; melting curve: 5 s, 65–95 °C. Relative transcription levels of *JASMONATE RESISTANT 1* (*JAR1, At2g46370*), *JASMONATE-ZIM-DOMAIN PROTEIN 10* (*JAZ10, At5g13220*) and *VEGETATIVE STORAGE PROTEIN* 2 (*VSP2, At5g24770*) were calculated using the ∆∆ Ct method (Pfaffl 2001) and normalized to their respective controls. *Actin2* (*At3g18780*) was used as housekeeping gene. All primer pairs used were described and characterized (Scholz et al. 2014).

### Volatile Collection and Analysis

Lima bean was used to study the volatile organic compound (VOC) emission of these plants. Therefore, the pots containing plants with fully developed primary leaves were wrapped in aluminum foil to avoid soil volatile collection. The plants were split again randomly into four groups and either exposed to *A. sexdens* ants, *S. littoralis* caterpillars, treated with scissors, or served as control. More precisely, plants used for *A. sexdens* treatment were placed in contact with the colony until sufficient ants accumulated on the leaves and first damages occurred. Then, the plant and its ants were transferred to a glass container that could be closed air-tied and volatiles were collected for 24 h, while the ants were removed after 5 h. For the caterpillar treatment, both, plants and caterpillars were placed directly into the glass container and volatiles were collected for 24 h, while the caterpillars were removed when they achieved as much damage as the ants (approx. 4 h). The wounding treatment resembled the ant damage in quantity and shape; however, it was conducted within 5 min, after which the 24 h volatile collection started. For the collection itself, a push-pull system was used, where charcoal purified air was pumped into the glass containers at a flow rate of 0.6 L min^− 1^ meanwhile 0.4 L min^− 1^ were pumped from the plant headspace out of the system passing through a 20 mg PoraPak (Alltech, Deerfield, IL, USA) filter that adsorbed the volatiles. At the end of the experiment, all leaves were excised, photographed, flash-frozen in liquid nitrogen, and freeze-dried to determine the dry weight of the leaves. The volatile compounds were eluted from PoraPak filters using 200 µL dichloromethane containing 10 ng µL^− 1^*n*-bromodecane (Sigma-Aldrich, Taufkirchen, Germany) as an internal standard. Samples were analyzed with GC-MS (gas chromatography – mass spectrometry) using a Hewlett-Packard model 6890 gas chromatograph (Agilent Technologies, Santa Clara, CA, USA) equipped with a Optima-5 column (30 m × 0.25 mm; 0.25 μm film thickness) (Macherey-Nagel, Düren, Germany) and quantified with GC-FID (flame ionization detector) using the methods described in Müller et al. (2022). The total volatile emission was calculated from all features present at concentrations of 15 ng/h/plant or higher in at least one of the samples. Compounds were tentatively identified with the library NIST17 and if possible by comparison to authentic standards.

### Statistical Analysis and data Visualization

**Fig. 1:**
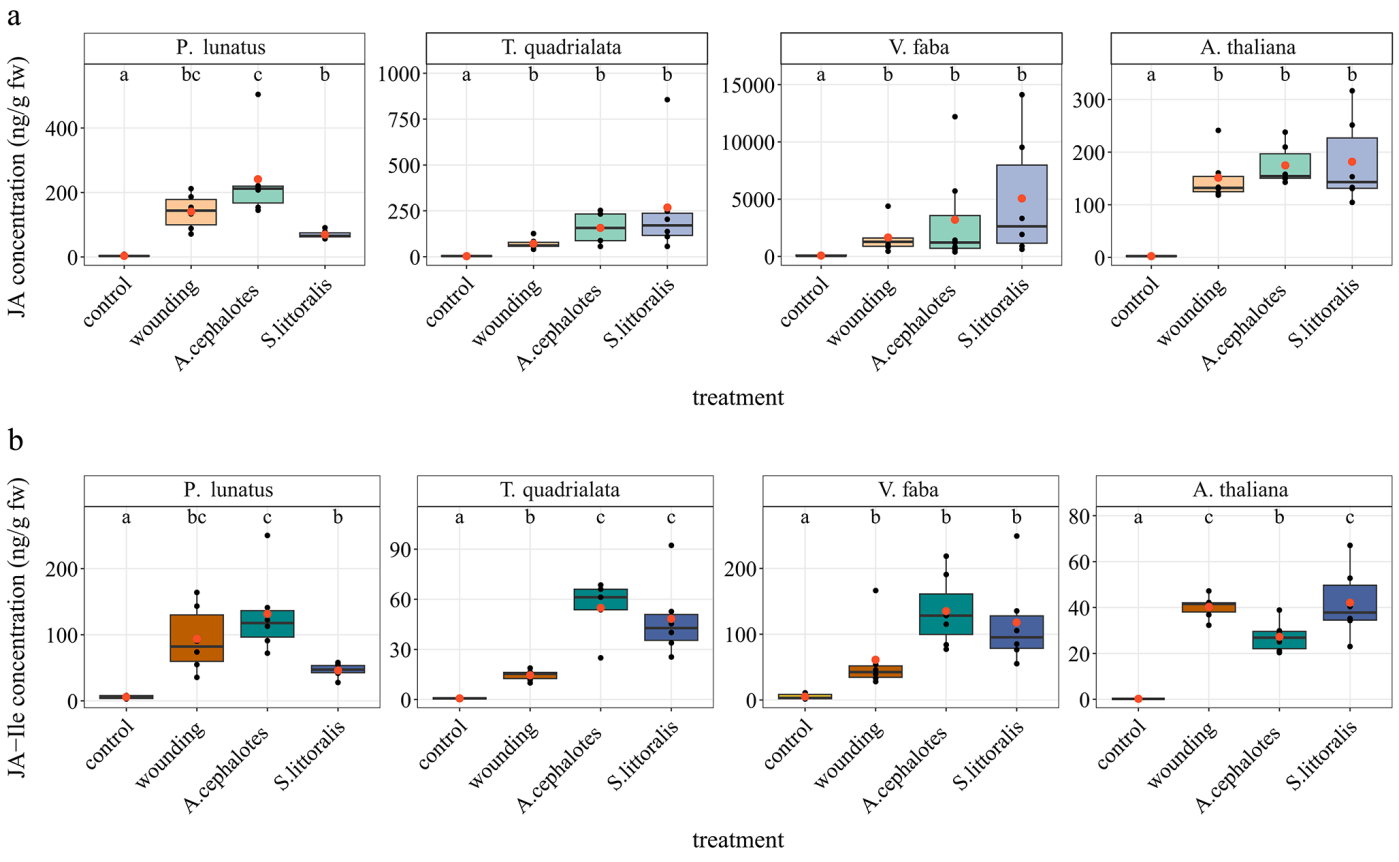
Jasmonate levels in different plant species in response to different modes of damage. (**a**) jasmonic acid (JA); (**b**) jasmonic acid-isoleucine conjugate (JA-Ile). Leaf-cutting ant (*Atta cephalotes*) damage was compared to other tissue damages such as feeding of generalist caterpillars (*Spodoptera littoralis*) or mechanical damage (“wounding”) for 1 h in *Arabidopsis thaliana*, *Vicia faba*, *Phaseolus lunatus*, and for 3 h in *Tococa quadrialata*. Leaves of untreated plants served as control. All data are presented as boxplots (center line, median; box limits, upper and lower quartiles; whiskers, 1.5x interquartile range; orange dot, mean). Statistical differences between treatments were analyzed by one-way ANOVA on ln-transformed data and Tukey HSD post hoc test (*n* = 4–6). Significant differences are indicated by different letters (*P* < 0.05)

**Fig. 2:**
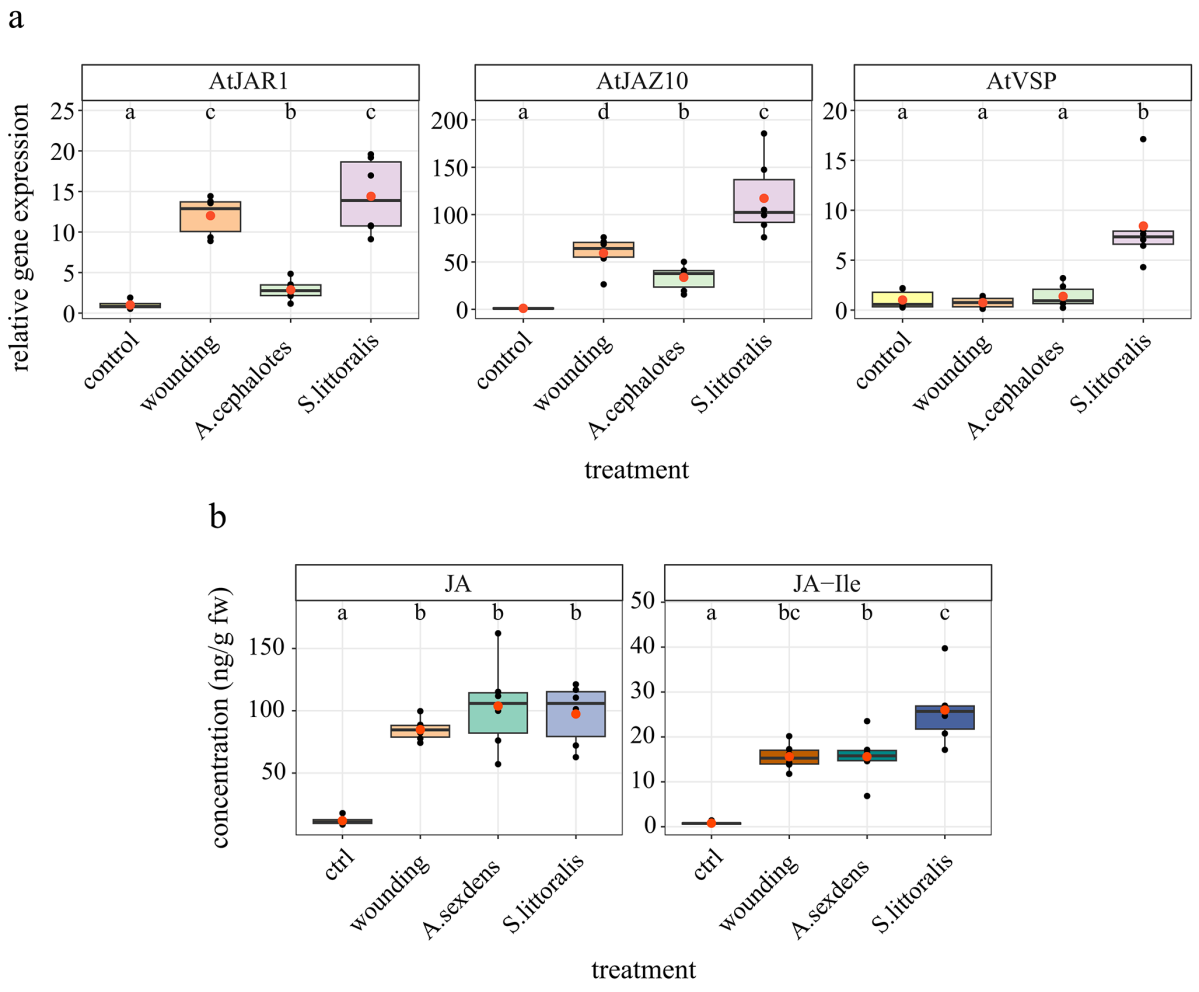
Analysis of jasmonate signaling in *Arabidopsis thaliana* upon various kinds of leaf damage. (**a**) Transcription levels of *JASMONATE RESISTANT 1* (*JAR1*), *JASMONATE-ZIM-DOMAIN PROTEIN 10* (*JAZ10*) and *VEGETATIVE STORAGE PROTEIN* (*VSP*) were analyzed by q-PCR in leaves after 1 h of different kinds of leaf damage, including *Spodoptera littoralis* larvae feeding, *Atta cephalotes* leaf cutting or mechanical damage (“wounding”) mimicking the ant leaf-cutting. Leaves of untreated plants served as control. Statistical differences between treatments were analyzed by one-way ANOVA on ln-transformed data and Tukey HSD post hoc test (*n* = 6). Significant differences are indicated by different letters (*P* < 0.05). (**b**) Jasmonate levels were analyzed in leaves that were subjected to the same treatments as described in (a), but with a different ant species (*A. sexdens* instead of *A. cephalotes*). All data is presented as boxplots (center line, median; box limits, upper and lower quartiles; whiskers, 1.5x interquartile range; orange dot, mean). Statistical differences between treatments were analyzed using one-way ANOVA on ln-transformed data and Tukey HSD post hoc test (*n* = 6). Significant differences are indicated by different letters (*P* < 0.05)

**Fig. 3:**
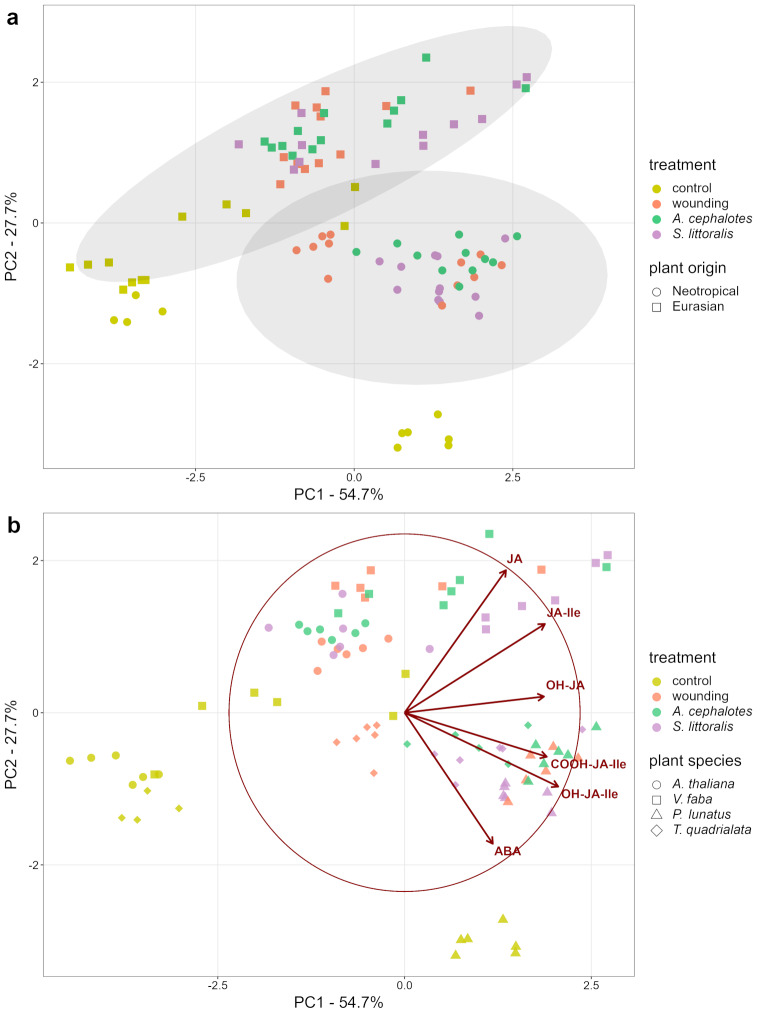
Volatile emission of *Phaseolus lunatus* upon different kinds of leaf damage. *Atta sexdens* ants or *Spodoptera littoralis* larvae were allowed to cut for 5 h or feed (to the same level of damage as the ants; ca. 4 h) on the leaves, respectively. Wounding with scissors mimicked the ant damage. Volatiles were collected continuously for 24 h, starting with the beginning of the respective treatment. Compounds were analyzed with GC-MS, tentatively identified with the library NIST17 and if possible by comparison to authentic standards. GC-FID analysis was used for quantification. The boxplots (center line, median; box limits, upper and lower quartiles; whiskers, 1.5x interquartile range; orange dot, mean) show the total amount of volatiles emitted by *S. littoralis*-damaged leaves (purple), *A. sexdens*-damaged leaves (green), mechanically wounded leaves (orange) and undamaged (ctr, yellow) leaves. Different letters indicate significant differences (*P* < 0.05), as determined by one-way-ANOVA and Tukey HSD post hoc analysis on log-transformed data (*n* = 5–6)

**Fig. 4:**
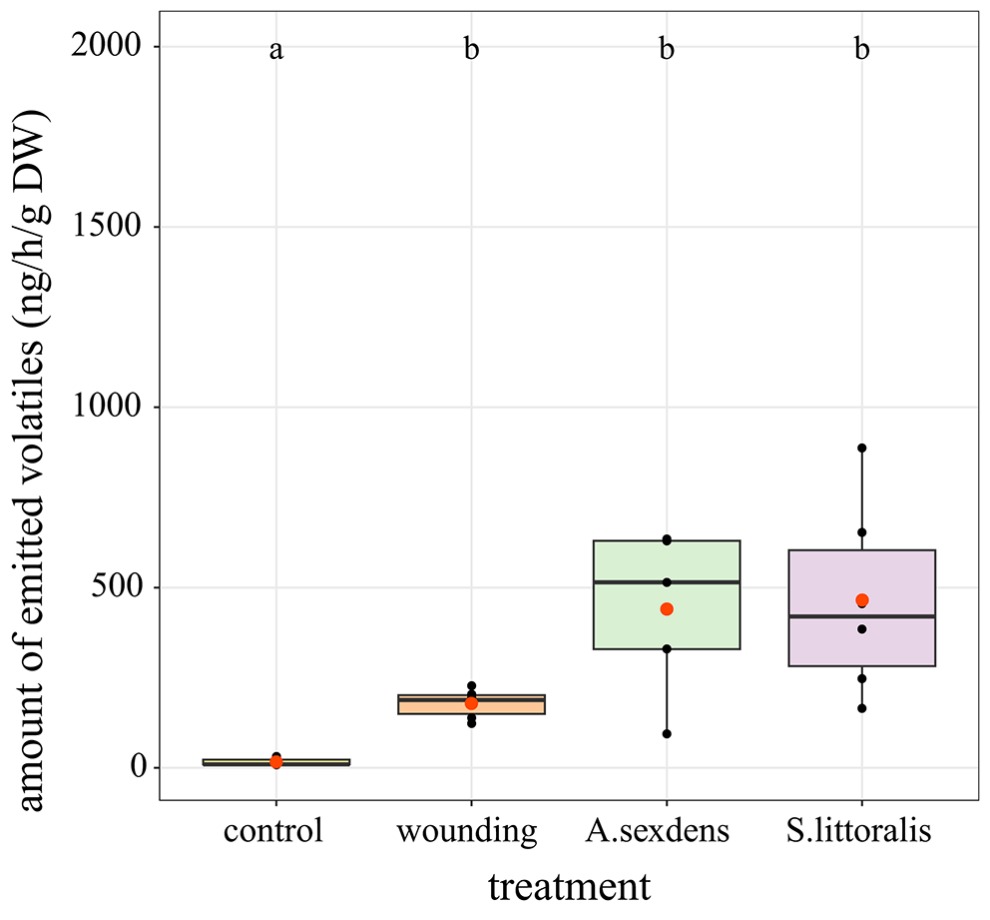
(**a**) Principal component analysis (PCA) scores plot of the defensive phytohormone profiles of Neotropical and Eurasian plants upon various kinds of leaf damage. Displayed are the first two principal components covering 82.4% of the variation of the dataset. 95% confidence ellipses were drawn regarding the species origin. The dataset was ln(x + 1)-transformed, scaled and centered. (**b**) Biplot of the PCA analysis above. The observations are displayed as transparent dots and the correlations of the analyzed variables (ABA, JA, JA-Ile, OH-JA, OH-JA-Ile, COOH-JA-Ile) with PC1 and PC2 are displayed as red arrows (scaled for visualization)

All statistical analysis were performed in R (version 4.3.1, R Core Team 2023) within R Studio environment (R Core Team 2022) or Sigma Plot (version 14.0, Systat Software, Inc.). We employed one-way ANOVA followed by a Tukey HSD post hoc test to determine if there are differences between the means of the inspected treatment groups that are caused by the applied treatments. This was conducted for every individual plot shown (Figs. 1, 2 and 3). The effect of the treatment or the differences between two groups were deemed significantly different when *P* < 0.05. Additionally, the statistical tests employed are mentioned in the figure legends and the results of these tests as well as the number of biological replicates per group are mentioned in the results section. All data was tested for statistical assumptions (normal distribution and homoscedasticity) by diagnostic plots and transformed by ln- or log-transformation if necessary. Principle component analysis was performed using the prcomp function of the R stats package. The data was ln-transformed and scaling to unit variation was applied to reduce the effects of outliers and variation of measurement sizes. The correlations of the variables with the principle components were calculated using the factoextra packages get_pca_var() function and plotted in Fig. 4b with a factor of 2.35 for visualization. Data visualization was performed using R and RStudio as mentioned. Details can be found in the figures and corresponding figure legends.

## Results

In order to analyze whether or not leaf cutting ants are recognized by their host plants and subsequently induce jasmonates and downstream defense responses, we used two Neotropical plant species (lima bean *(P. lunatus)*, tococa (*T. quadrialata*)) and two plant species with origin from Eurasia (faba bean (*V. faba)*, Arabidopsis *(A. thaliana)*) for the experiments.

### Induced Jasmonate Signaling

Both JA, as well as the bioactive jasmonate, JA-Ile, have been analyzed upon different treatments, i.e. *A. cephalotes* attack, wounding that mimicked *A. cephalotes* cutting, and feeding of herbivorous *S. littoralis* larvae. Compared to the non-damaged controls, in all tested plant species JA was significantly induced upon all treatments (Fig. 1a). The same holds true for JA-Ile (Fig. 1b) (one-way ANOVA on ln-transformed data; JA: *A, thaliana*: *F*_3,20_ = 331.7, *P* < 0.001; *T. quadrialata*: *F*_3,19_ = 34.29, *P* < 0.001; *P. lunatus*: *F*_3,20_ = 129, *P* < 0.001; *V, faba*: *F*_3,21_ = 15.73, *P* < 0.001; JA-Ile: *A, thaliana*: *F*_3,20_ = 648.1, *P* < 0.001; *T. quadrialata*: *F*_3,19_ = 111.9, *P* < 0.001; *P. lunatus: F*_3,20_ = 57.87, *P* < 0.001; *V, faba*: *F*_3,21_ = 43.18, *P* < 0.001; *n* = 4–6). Furthermore, the amount of JA accumulation upon the wounding, *S. littoralis* and *A. cephalotes* treatments was similar in all plant species examined. Interestingly, the pattern of JA-Ile accumulation was slightly different: JA-Ile accumulation upon *A. cephalotes* attack was similar to either mechanical damage or *S. littoralis* treatment in all plants except for *Arabidopsis*. Here, upon *A. cephalotes* leaf cutting the level of JA-Ile was significantly lower when compared to wounding or *S. littoralis* feeding (Fig. 1b) (Tukey HSD: *P* < 0.05). This particular result for *Arabidopsis* was further supported by qPCR analysis of herbivory induced, defense-related genes (Fig. 2a). *JAR1* and *JAZ10* gene expression was significantly higher after all treatments when compared to the control but mRNA accumulation after *A. cephalotes* cutting was significantly lower in comparison with wounding or *S. littoralis* feeding (one-way ANOVA on ln-transformed data; *AtJAR1*: *F*_3,20_ = 63.482, *P* < 0.001; *AtJAZ10*: *F*_3,20_ = 143.988, *P* < 0.001; *AtVSP2*: *F*_3,20_ = 11.109, *P* < 0.001; *n* = 6). Induced *VSP2* gene expression was found only after larval herbivory. To investigate if the leaf-cutting ant effect on JA-Ile in *Arabidopsis* was due to the particular ant species involved, we employed another *Atta* species, *A. sexdens*, and repeated the experiment, again with different treatments and subsequent phytohormone analysis. As shown in Fig. 2b, the results obtained were similar to the initial experiment. *A. sexdens* cutting induced a significant increase of JA and JA-Ile (one-way ANOVA on ln-transformed data: JA: *F*_3,20_ = 92.72, *P* < 0.001; JA-Ile: *F*_*3,20*_ = 146.5, *P* < 0.001; *n* = 6), however, JA-Ile levels were significantly lower compared to *S. littoralis* feeding (Tukey HSD: *P* < 0.05).

The mixed results of the JA-Ile response to treatments in the four plant species prompted us to further explore the phytohormone profile (jasmonates, abscisic acid (ABA)) of these plant species. Figure 4a shows the PCA (principal component analysis) score graph, where the principal components (PC) 1 and 2 explained 82.4% of the total variance of the dataset. Samples formed distinct clusters based on their received treatments, as well as the phylogenetic and the geographic origins of the plant species. More precisely, control groups separated from all of the tissue-damaging treatments (mechanical wounding, *S. littoralis*, or *A. cephalotes* herbivory). Additionally, there was a clear sample separation into a Neotropical (*P. lunatus* and *T. quadrialata*) and an Eurasian (*A. thaliana* and *V. faba*) group, respectively (Fig. 4a). This separation was mainly evident along PC2, which accounted for 27.7% of the total variance. According to correlation values of the variables with the PCs, this separation is caused by the positive and negative correlation of PC2 with JA and ABA respectively (Fig. 4b). In all damage treatments, the samples cluster according to their respective plant species (Fig. 4b). For controls, the samples of the Neotropical plant *T. quadrialata* and the two Eurasian plants *A. thaliana* and *V. faba* overlap while samples of *P. lunatus* form a distant cluster.

### Plant Volatile Emission

Kost and colleagues (2011) published the only study focusing on jasmonates and leaf-cutting ants. Using *A. colombica* ants and *P. lunatus* as host plant their results suggested that after a singular treatment neither leaf-cutting ants nor wounding can induce noteworthy VOC emission, in contrast to herbivory or JA treatment. Because we detected reasonable levels of jasmonates already after 1 h of treatment (Fig. 1), we decided to repeat the VOC experiment with *P. lunatus* and a slightly modified experimental approach. Strikingly, after a singular treatment with *A. sexdens* or mechanical wounding, a significant VOC production and emission was detected compared with the non-treated control, although wounding caused lower and much less diverse VOC emission (Fig. 3) (one-way ANOVA on log-transformed data (*F*_3,18_ = 36.94, *P* < 0.001; *n* = 5–6) and TukeyHSD post hoc analysis (*P* < 0.05). Upon insect treatments, among the detected compounds we identified 14 different VOC, including several fatty acid derivatives (green leaf volatiles): (*E*)-3-hexenal, (*E*)-3-hexen-1-ol, 1-hexanol, 1-octen-3-ol, octan-3-one, *cis*-3-hexenyl acetate, *cis*-3-hexenyl-α-methyl-butyrate, (*Z*)-3-hexenyl-(*E*)-2-methyl-but-2-enoate; mono-, sesqui-, and homoterpenoids: (*E*)-β-ocimene, linalool, α-farnesene, (*E*)-4,8–dimethyl–nonatriene (DMNT); and indole (Fig. S2).

## Discussion

Plants usually recognize attacking herbivores and react accordingly to defend themselves (Maffei et al. 2007; Mithöfer and Boland 2012). A special situation is given if foraging leaf-cutting ants are the aggressors because they do not feed on the host plant but cut off leaf pieces and carry them away to their nests (Wirth et al. 2003). The cutting speed varies for each leaf type; young and thin leaves are cut at a faster average rate than mature and thicker leaves (Burd 1996). Physical features in addition to leaf density (e.g., cuticle thickness, trichome density or thickness) are likely to affect the cutting speed (Howard 1988). On average, the total time used to cut off a leaf fragment (for *A. cephalotes, A. colombica, A. sexdens*) is between 2 and 7 min, ranging from less than 1 to > 10 min, while the mean cutting speed is between 0.08 and 0.24 mm sec^− 1^ (Roces and Hölldobler 1994; Roces and Lighton 1995; Burd 1996). Thus, leaf-cutting ants never stay long on the leaves of interest and therefore may undermine some elements of the host plants’ induced defense.

Little is known about the metabolic changes caused in plants attacked by leaf-cutting ants. In the present study, we asked whether the ants’ leaf harvesting behavior is sufficient to trigger rapid, typical defense reactions in the plants.

Nearly all herbivory- and wounding-triggered defenses are based on the induction of jasmonates (Maffei et al. 2007). As this topic has never been studied in plants attacked by leaf-cutting ants, we first focused on assessing these phytohormone concentrations in comparison to mechanical wounding as well as herbivory by feeding *Spodoptera littoralis* larvae. In all tested plant species, herbivory by leaf-cutting ants, caterpillars, or mechanical wounding led to the induction of jasmonates (Fig. 1), although the strength of induction varied between plant species and treatment.

Insect feeding is a combination of tissue wounding and the introduction of insect-derived oral secretion (OS) during the feeding process (Mithöfer and Boland 2008). The OS might contain signaling compounds, so-called herbivore-associated molecular patterns (HAMP), which can be recognized by the plant. Mimicking larval feeding by a robotic device showed that continuous mechanical wounding alone is sufficient to induce classical defense response such as VOC emission (Mithöfer et al. 2005). However, the simultaneous addition of OS modulates the plant defense response to a certain extent and the induced defense responses are even more similar to responses elicited by insect herbivory (Li et al. 2019). For leaf-cutting ants, it is not known if they provide OS or any other chemical compounds while they cut off the leaf tissue. The former is unlikely because leaf-cutting ants do not ingest the plant material and their mandibles only slide across the leaf blade during cutting (Silva et al. 2016). This way of cutting seems to be similar to the wounding we did with scissors. Nevertheless, *Atta* species are known to possess mandibular gland secretion (Rodrigues et al. 2008). It is therefore conceivable that fluid from the ants’ mandibular glands is exposed to plant tissue. These secretions could explain the results we found in *A. thaliana.* Leaves that were attacked by *A. cephalotes* ants induced lower levels of JA-Ile compared to mechanical wounding and the feeding of *S. littoralis* caterpillars (Fig. 1b). Interestingly, this stands as an exception to the other plant species examined but it is supported by similar findings in the repetition of the experiment with *A. sexdens* ants (Fig. 2b). Additionally, further qPCR-based analysis of the defense-related marker genes *AtJAR1* and *AtJAZ10*, supported this hypothesis. The induction of the relative gene expression for these two genes was significantly lower in leaves attacked by *A. cephalotes* than in leaves subjected to mechanical wounding or *S. littoralis* caterpillar feeding (Fig. 2a). This may indicate a suppression of the defense mediation by signals derived from the attacker, which is an already known mechanism for herbivores (Kant et al. 2015). Interestingly, the expression of another defense-related marker gene, *AtVSP2*, was only induced upon *S. littoralis* feeding. This gene is known to respond more slowly and is often not detectable within the first hour after the beginning of the treatments (Berger et al. 2002). During feeding, the caterpillars bring the freshly wounded plant tissue into contact with their OS and thus with previously eaten and partially digested plant fragments. As this way of feeding is more intimate than the cutting behavior of leaf-cutting ants, this could reason the differences of the induced relative expression of *AtVSP2* that we observed in *A. thaliana* leaves.

As neither *A. thaliana* nor *V. faba* are natural host plants for leaf-cutting ants, these species may respond differently to *Atta* attacks compared with Neotropical plant species such as *P. lunatus* and *T. quadrialata*. Thus, we performed a PCA of defense-related phytohormone compositions of the two Neotropical and two Eurasian species upon the different leaf damaging treatments. Besides jasmonates, we included abscisic acid (ABA) in the analysis (Fig. 4). It is well known that leaf damage can cause water loss by creating open wounds, which increase transpiration (Ostlie and Pedigo 1984; Aldea et al. 2005). This water loss subsequently triggers the accumulation of the drought-associated phytohormone ABA (Lim et al. 2015). In addition, ABA is directly involved in herbivory-induced defenses (Peña-Cortes et al. 1989; Vos et al. 2013). We identified JA and especially ABA as strong factors, explaining this result (Fig. 4b). Our analysis further revealed that the data sets obtained for the different plant species do not separate by the experienced types of damage. Instead, clusters are formed based on the species itself. This suggests that all four tested species react somehow individually to the applied treatments. However, none of them differed drastically in their response to wounding by leaf-cutting ants compared to other damage treatments. Interestingly, even *P. lunatus* and *V. faba* form very distant clusters with no observed overlap, although both species belong to the same taxonomic family of Fabaceae. On the other hand, we observed a subdivision of plant species according to their geographical origin into Neotropical and Eurasian species (Fig. 4a). These unexpected results suggest that, at least in our study, it is the origin of a plant species rather than its taxonomic relationship that determines physiological stress responses. Taken together, these findings open a window to explore the defense signaling and response of plant species to herbivory in relation to or depending on their environment, their geographic and their genetic origin. Further studies including more species from a diverse set of origins which covers taxonomically related and non-related species could verify or falsify our hypothesis and findings and may provide clues regarding the nature and evolution of particular plant defense-related reactions and features.

The emission of VOCs is a typical plant defense response to herbivory (Kant et al. 2009; Baldwin 2010). How the plant volatile emission upon an attack of leaf-cutting ants compares to an attack of chewing herbivores is largely unknown. *Acromyrmex rugosus* attacks induced both changed density of leaf trichome morphotypes and changed VOC emission in subsequently grown leaves of the aromatic plant *Ocimum gratissimum* (Tozin et al. 2017). Whether or not the detected VOC contributed significantly to the plant’s defense remained an open question of that study. However, previous studies indicated that the emitted (E)-ß-ocimene and ß-carophyllene as well as other terpenoids are repellent to *Atta* workers (Chen et al. 1984; Hubbell et al. 1983; Howard et al. 1988). Similarly, *P. lunatus* leaves exposed to *A. colombica* also emitted significantly more VOC than control plants when the leaf damage was stepwise increased over three consecutive days (Kost et al. 2011). However, a single wounding event or a fast leaf tissue removal (within 20 min) by *Atta* species was not sufficient to induce VOC (Kost et al. 2011). This is in accordance with the findings of Mithöfer et al. (2005) showing that in *P. lunatus* the VOC induction results from continuous wounding over time, not a single event.

Here, we analyzed the volatile emission of lima beans upon an attack of *Atta sexdens* for 24 h and compared it to mechanical wounding as well as feeding of *S. littoralis*. All treatments led to the emission of volatile compounds, including wounding associated C_6_ and C_8_ volatiles as well as JA-associated volatiles like indole (Fig. 3; Fig. S2). This is in contrast to the previously mentioned studies of Kost et al. (2011), which did not find a significant induction of VOC after a single, fast attack of *Atta colombica*. These differences might be explained by varying times of treatment and handling procedures. In the study of Kost and coworkers (2011), the ants cut 20% of the leaf area within 20 min before they were removed and the volatile collection was started. In our experiments, the ants had access to the plants for 5 h, and volatiles were collected throughout this time span to capture the whole volatile spectrum, including VOC emitted from the cut-off plant parts. The emission of green leaf volatiles like hexen-1-yl acetate analyzed in both studies was shown to peak 5 min after a wounding event and to return to base line levels 15 min afterwards (D’Auria et al. 2007). Our approach allowed us to collect these volatiles whereas we can only speculate that the other approach missed this first response. Combined, both studies illustrate the importance of the effective wounding time, whereas the area separated from the leaf seems to be of secondary importance.

An important ecological feature that distinguishes the interaction between plants and leaf-cutting ants from almost all other studies on induced plant defense is that the insect does not eat the plant tissue, but feeds it to mutualistic fungi. This raises the question of whether and how the changes in plant defense observed in our study have a further effect. Two levels of interaction must be considered here, firstly that between the plants and the ants and secondly that between the ants and the fungi. Previous studies have shown that *Atta cephalotes* ants avoid plants with certain constitutively present and defense-related secondary metabolites (Littledyke and Cherrett 1978; Chen et al. 1984; Hubbell et al. 1983; Howard 1988; Howard et al. 1988). The reason for avoidance may be a deterrent or toxic effect of the defense metabolites on the ants. Thus, we postulate this may also apply to inducible metabolites. As soon as such compounds have accumulated, the ants avoid these plants as suggested by the results of Kost et al. (2011), and would start searching for a new host plant. This scenario may also explain why leaf-cutting ants rarely completely defoliate a plant (Cherrett 1968; Wirth et al. 2003). In addition, secondary metabolites induced in the harvested plants may also have an impact on the fungi that are supplied with such plant material. In the worst case scenario for the ants, such metabolites could be toxic to the fungi but not to the ants. This would be dramatic for the ant colony. To avoid this danger, the ants would have to take into account any kind of change in the metabolite composition in the host plant and stop collecting. Furthermore, it is to be expected that there is a kind of feedback loop that informs the ants about the condition of the fungi in the nest and allows them to change the host plant in critical situations. Studies in this regard could be carried out with e.g. previously jasmonate-induced plants.In conclusion, in our experiments we found limited evidence that herbivory by leaf-cutting ants differs from caterpillar derived herbivory or mechanical wounding. There were no differences in the induction of jasmonates (except *A. thaliana*) in the investigated plant species, or in the emission of herbivore-induced plant VOCs by lima bean. However, exploratory data analysis hinted to differences in the defense phytohormone reaction between Neotropical and Eurasian plants, with JA and ABA as main determinants.

## Electronic Supplementary Material

Below is the link to the electronic supplementary material.

Supplementary Material 1

## Data Availability

The data that support the findings of this study are available in the supplementary material of this article. Any additional information will be provided by the corresponding author upon reasonable request.
