# Supplementary material for: Herbivory by Leaf-Cutting Ants: Exploring the Jasmonate Response in Host and Non-Host Plants
Source: J Chem Ecol. 2024 Jun 20;50(11):767–77. doi: 10.1007/s10886-024-01519-5 (PMC11543716; doi:10.1007/s10886-024-01519-5)
Supplement: Supplementary file 1 — Supplementary Material 1 [file 10886_2024_1519_MOESM1_ESM.pdf]

Supplemental Information

Herbivory by Leaf-cutting Ants: Exploring the Jasmonate Response in Host and non-Host Plants

ANDREA TERESA MÜLLER[1†], KILIAN LUCAS OSSETEK[1†], AXEL MITHÖFER[1*]

[1]Research Group Plant Defense Physiology, Max Planck Institute for Chemical Ecology, Jena, Germany

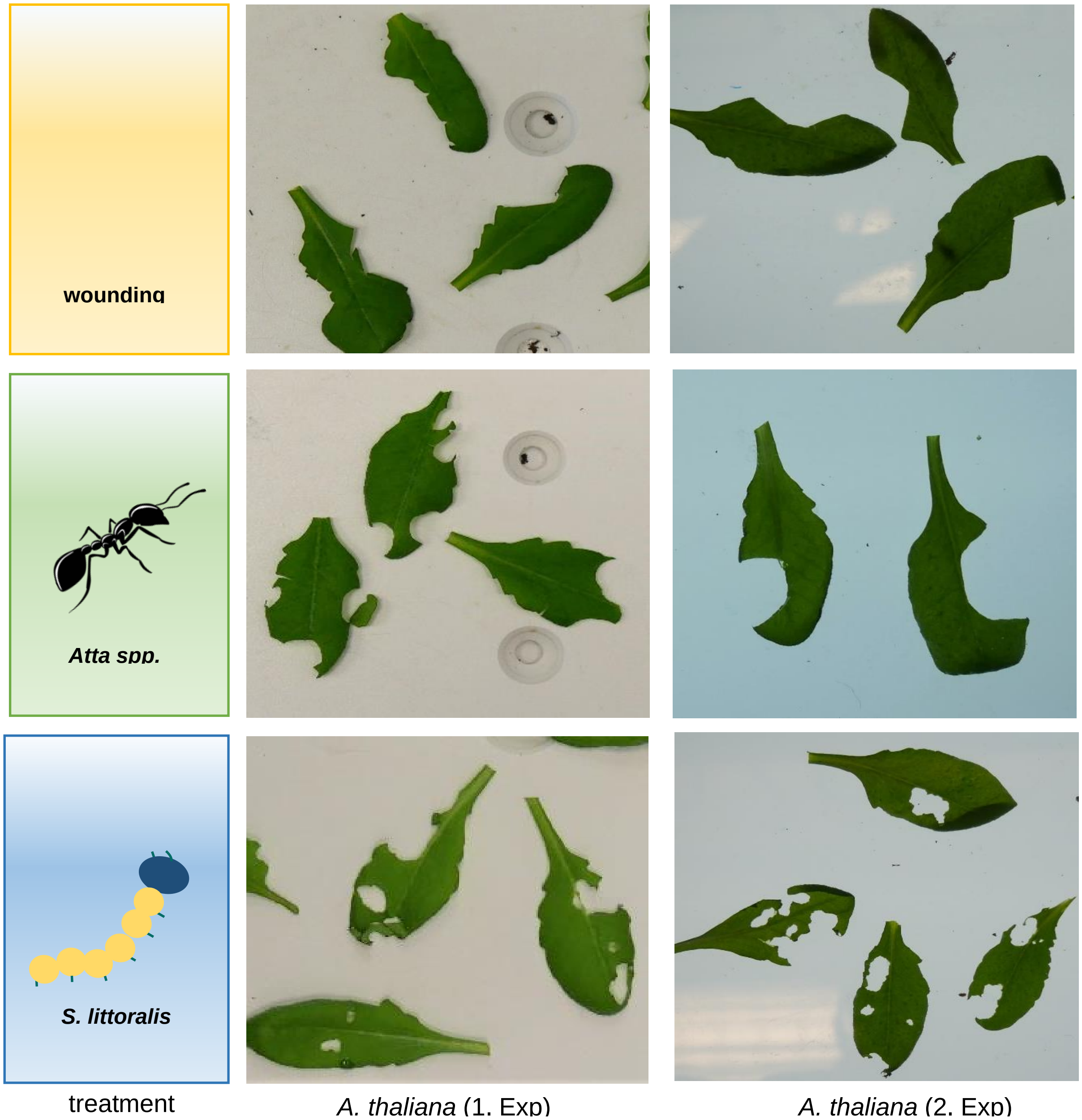

Fig. S1 Comparison of the three different treatments on Arabidopsis and documentation of remaining leaves used for further experiments.

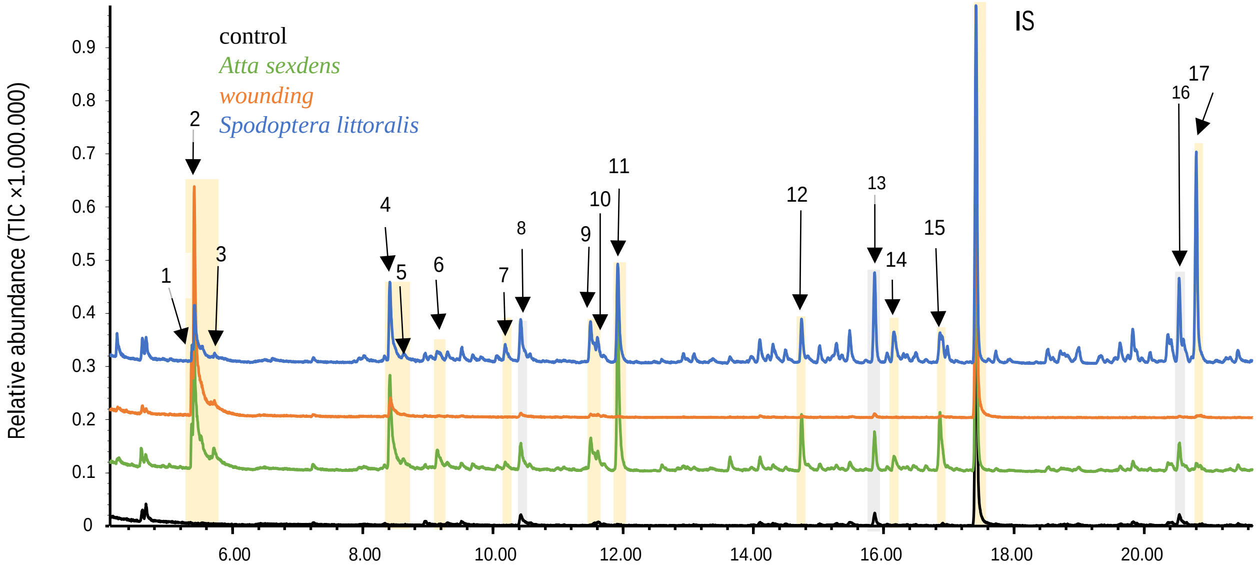

Fig. S2 Volatile profiles of lima bean (*Phaseolus lunatus*) upon different kinds of leaf damage. Comparison of the volatile bouquets of *Spodoptera littoralis*-damaged leaves (blue), *Atta sexdens*-damaged leaves (green), mechanically wounded leaves (orange) and undamaged (ctr, black) leaves. *S. littoralis* larvae or *A. sexdens* ants were placed on the plants and allowed to feed on/cut the leaves for **4-5 h** while the volatiles were collected simultaneously. After that time, the insects were removed and the volatiles collected for additional **19-20 h**. To mimic the ants, leaves of the third group were damaged with scissors and volatiles collected for 24 h. Compounds were analyzed with GC-MS and identified with the library NIST17 and if possible (numbers in bold) by comparison to authentic standards. IS: internal standard; c: contamination, **1**: (*E*)-3-hexenal, **2**: (*E*)-3-hexen-1-ol, **3**: 1-hexanol, **4**: 1-octen-3-ol, **5** octan-3-one, **6**: *cis*-3-hexenyl acetate, **7**: (*E*)-β-ocimene, 8: unknown, **9**: linalool, **10**: nonanal, **11**: (*E*)-4,8–dimethyl–nonatriene (DMNT), **12**: *cis*-3-hexenyl-α-methyl-butyrate, 13: unknown, **14**: indole, **15**: (*Z*)-3-hexenyl-(*E*)-2-methyl-but-2-enoate, 16: unknown, **17**: α-farnesene, TIC: total ion chromatogram.
